# Systematic structure-based analysis of RET variants in MEN2A and Hirschsprung's disease, and the paradoxical co-occurrence of both conditions

**DOI:** 10.1242/dmm.052748

**Published:** 2026-04-27

**Authors:** Anna Fassler Bakhman, Michal Cohen, Rachel Kolodny, Mickey Kosloff

**Affiliations:** ^1^ Department of Human Biology, Faculty of Natural Science, University of Haifa, Haifa 3103301, Israel; ^2^ Pediatric Endocrinology Unit, Ruth Rappaport Children's Hospital, Rambam Health Care Campus, Haifa 3109601, Israel; ^3^ The Ruth & Bruce Rappaport Faculty of Medicine, Technion-Israel Institute of Technology, Haifa 3525433, Israel; ^4^ Department of Computer Science, University of Haifa, Haifa 3103301, Israel

**Keywords:** Receptor tyrosine kinase, Cancer, Endocrine tumors, Multiple endocrine neoplasia, Hirschsprung's disease, Human genetic variants

## Abstract

Variants in the human receptor tyrosine kinase RET can cause RET loss-of-function and Hirschsprung's disease (HSCR), while activating *RET* variants drive cancers including multiple endocrine neoplasia type 2 (MEN2). Paradoxically, some variants cause both HSCR and MEN2A. We curated 77 RET extracellular positions associated with HSCR, MEN2A or both and used a structure-based approach to predict the effects of variants at these positions on RET structure. Approximately 90% of HSCR-associated positions can, upon mutation, disrupt intramolecular interactions stabilizing RET tertiary structure via distinct mechanisms. Only a minority perturb protein−protein interactions needed for signal activation. In contrast, our analysis showed that ∼75% of variants causing MEN2A lead to an unpaired cysteine that can form an intermolecular disulfide bond between two RET monomers. Other MEN2A variants are likely to enhance RET homodimerization via membrane-proximal extracellular interactions. Substitutions that, concurrently, destabilize RET structure and result in an unpaired cysteine are predicted to cause the paradoxical co-occurrence of HSCR and MEN2A. Our findings lay out a mechanistic basis for almost all identified pathological *RET* mutations, and suggest therapeutic strategies for targeting RET activity in HSCR and MEN2A.

## Teaser

**Summary:** Resources and mechanistic insights into how hereditary RET variants cause two orthogonal diseases – Hirschsprung's disease (HSCR) and multiple endocrine neoplasia type 2 (MEN2) syndromes – as well as how some variants paradoxically lead to both diseases.

## INTRODUCTION

The receptor tyrosine kinase *RET* gene encodes a transmembrane protein that belongs to the receptor tyrosine kinase family. RET initiates signaling pathways that regulate cell growth, differentiation and survival in diverse tissues including the embryonic kidney, the thyroid and the enteric nervous system (Schuchardt et al., 1994; Durbec et al., 1996a; Takahashi, 2022). Abnormal RET signaling can lead to disease, including Hirschsprung's disease (HSCR) – a congenital malformation characterized by segmental enteric agangliosis, which is often diagnosed in newborns and infants (Swenson, 2002; Anders et al., 2001; Takahashi, 2001). By contrast, sustained activation of RET signaling contributes to tumorigenesis and, in particular, to multiple endocrine neoplasia type 2 (MEN2) syndromes that typically present later in life (Amiel et al., 2008; Santoro and Carlomagno, 2013; Li et al., 2019; Regua et al., 2022). MEN2A, the most common type of MEN2, is characterized in the vast majority of affected individuals by the development of medullary thyroid carcinoma (MTC) or familial medullary thyroid carcinoma (FMTC) often presenting in the first to third decade of life. Additional manifestations of MEN2A can include pheochromocytoma (PHEO) or primary hyperparathyroidism (PHPT) and, rarely, also cutaneous lichen amyloidosis (CLA) (Eng and Plitt, 1999; Mathiesen et al., 2022). Notably, genetic abnormalities in *RET* have also been associated with conditions other than HSCR and MEN2, including papillary thyroid carcinoma, non-small cell lung cancer and congenital anomalies of the urinary tract. However, these *RET* abnormalities typically involve only the intracellular domains of the protein (Nikiforov, 2002; Davis et al., 2014; Prescott and Zeiger, 2015; Andrini et al., 2022; Novello et al., 2023).

**Fig. 1.**
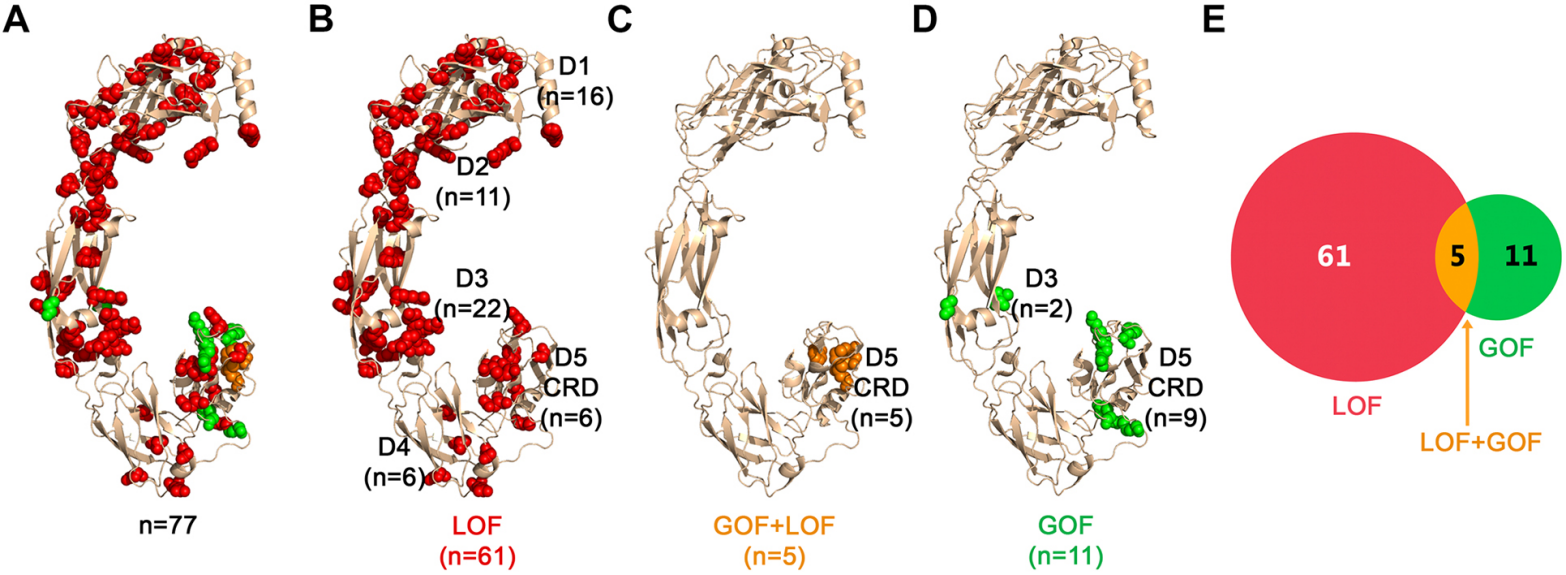
**Positions in the extracellular side of RET that are associated with HSRC and/or MEN2A.** (A) GOF and LOF variants across 77 RET positions that are associated with HSRC and MEN2A, respectively. Positions that are linked only with HSRC (LOF) are shown as red spheres, positions linked only to MEN2A (GOF) are shown as green spheres and positions associated with both diseases are shown as orange spheres. (B) LOF positions associated with HSRC only, colored as in A. (C) ‘Janus’ mutations that are associated with both HSRC (LOF) and MEN2A (GOF), colored as in A. (D) GOF positions associated only with MEN2A, colored as in A. (E) A Venn diagram showing the number of the LOF- and GOF-associated positions as shown in panels A-D.

The RET receptor activates downstream signaling cascades via PI3K/AKT, RAS/RAF/MEK/ERK, JAK2/STAT3 and PLCγ (Regua et al., 2022). RET activation is typically initiated by binding of any of the following four ligands: glial cell line-derived growth factor (GDNF), neurturin (NRTN), artemin (ARTN) or persephin (PSPN) – usually combined with binding to one of four GDNF family receptor alpha (GFRa) co-receptors (i.e. GFRa1, GFRa2, GFRa3, GFRa4) (Durbec et al., 1996b; Kotzbauer et al., 1996; Baloh et al., 1998). X-ray structures of RET, solved in complex with these ligands and co-receptors, have demonstrated that the extracellular part of the RET receptor is composed of four (D1-D4) cadherin-like domains, followed by one cysteine-rich domain (CRD) designated D5 (Li et al., 2019; Goodman et al., 2014; Bigalke et al., 2019) (Fig. 1). In the tetrameric complex of RET with GFRα2 and NTRN, all five extracellular RET domains interact with either ligands or co-receptors (Li et al., 2019; Goodman et al., 2014), while in the dimeric complexes of RET with ligands and co-receptors only four domains (D1-D3, D5) contribute to these interactions (Bigalke et al., 2019). The structure of the tetrameric complex has further shown that the hexameric 2:2:2 NRTN−GFRα2−RET complex can dimerize, forming a 4:4:4 complex that suppresses RET endocytosis. This suggested that the RET receptor has two distinct interfaces with ligands and two distinct interfaces with its co-receptors.

The clinical relevance of the RET extracellular region has been demonstrated in studies that mapped gain-of-function (GOF) variants in cysteine residues located within the RET CRD, leading to MEN2 syndromes by triggering RET dimerization without requiring ligand and co-receptor binding (Chappuis-Flament et al., 1998; Kjaer et al., 2006; Asai et al., 1995). Such cysteine mutations, presumably, result in an unpaired cysteine that induces ligand-independent dimerization and constitutive activation of RET by forming an inter-chain disulfide bond (Li et al., 2019; Chappuis-Flament et al., 1998; Asai et al., 1995). In contrast, loss-of-function (LOF) variants in the RET extracellular region can lead to HSCR disease − presumably, by affecting the stability of the RET protein – thus, reducing functional enteric ganglia in the gut tissue (Takahashi, 2001; Li et al., 2019; Manie et al., 2001; Iwashita et al., 1996; Carlomagno et al., 1996). Paradoxically, four RET positions associated with with ‘Janus mutations’ – i.e. mutations that exhibit opposing functional effects – in cysteine (C) residues C609, C611, C618 and C620 have been shown to lead to both LOF and GOF effects, and co-occurrence of HSCR as well as MEN2A in the same individual or family (Takahashi, 2001; Mulligan et al., 1994; Borst et al., 1995; Pelet et al., 1998; Decker and Peacock, 1998; Takahashi et al., 1999; Nishikawa et al., 2003; Arighi et al., 2004; Kim et al., 2006).

In this current study, we computationally analyzed structures of the extracellular regions of RET, seeking mechanistic explanations for missense mutations that lead to HSCR, MEN2A or the paradoxical occurrence of both diseases. To this end, we identified which positions may perturb RET stability or protein−protein interactions upon mutation and which positions might form intermolecular bonds that can lead to constitutive activation. Our findings map 77 RET positions that have been reported to lead to clinical phenotypes of HSCR or MEN2A and predict the potential structural effects of mutations in all 594 positions that span RET extracellular regions. These insights suggest potential therapeutic avenues for particular variants, such as stabilizing RET structure for HSCR-associated positions or inhibiting aberrant dimerization with covalent inhibitors to specific cysteine residues for some MEN2A-associated positions.

## RESULTS

### Dataset of RET mutations that lead to diseases

We collected a dataset of 77 positions in the extracellular region of RET, in which missense mutations are documented as linked with HSRC and/or MEN2A (Table 1). To this end, we searched the ClinVar database (Landrum et al., 2014) for ‘pathogenic’ and ‘likely pathogenic’ *RET* variants associated with HSRC disease or MEN2A, and found 19 HSRC-associated RET positions and seven MEN2A-associated positions. One of these positions, phenylalanine (F)555, is associated with both diseases. A search in the COSMIC and cBioPortal databases did not identify additional positions of pathological relevance. We also searched the literature for additional RET positions reported to lead to HSRC or MEN2A (Anders et al., 2001; Pelet et al., 1998; Hofstra et al., 2000; Kashuk et al., 2005; Castellone et al., 2010; Castellone and Melillo, 2018; Krampitz and Norton, 2014). When the pathological classification of specific positions conflicted between ClinVar and the literature, we prioritized the latter. Specifically, some substitutions in positions C609, C611, C618 and C620 are classified in ClinVar as only GOF mutations (i.e. associated only with MEN2A), while the literature reports the same substitutions as leading to both HSRC and MEN2A (Pelet et al., 1998; Decker and Peacock, 1998; Nishikawa et al., 2003; Wells et al., 2015). Of the 77 RET positions we collected, 61 are linked exclusively to HSCR disease, 11 are associated only with MEN2A, and five Janus mutations are linked to both HSCR and MEN2A.

**Table 1.** List of 77 RET positions, whose missense variants are linked to HSCR and/or MEN2A

RET position	RET domain	Reported mutation	Reported disease	Source
S32	D1	S32L	HSCR	ClinVar VCV000013923.5; Li et al., 2019
Y36	D1	Y36C	HSCR	Lorente-Ros et al., 2020
L40	D1	L40P	HSCR	Li et al., 2019; Yin et al., 1994
L56	D1	L56M	HSCR	ClinVar VCV000036723.76; Hofstra et al., 2000
R57	D1	R57W	HSCR	Kashuk et al., 2005
P64	D1	P64L	HSCR	ClinVar VCV001782480.2; Li et al., 2019
L72	D1	L72P	HSCR	Lorente-Ros et al., 2020
R77	D1	R77C	HSCR	ClinVar VCV001069381.8; Li et al., 2019
G93	D1	G93S	HSCR	ClinVar VCV003065760.1; Li et al., 2019
Y96	D1	Y96C	HSCR	Kashuk et al., 2005
H114	D1	H114C	HSCR	Li et al., 2019; So et al., 2011
L123	D1	L123F	HSCR	Lorente-Ros et al., 2020
C142	D1	Unknown	HSCR	Li et al., 2019
A143	D1	A143G	HSCR	Lorente-Ros et al., 2020
V145	D1	V145G	HSCR	Li et al., 2019; So et al., 2011
F147	D1	F147S	HSCR	ClinVar VCV000599631.3
P155	D2	P155L	HSCR	Li et al., 2019; So et al., 2011
C157	D2	C157Y	HSCR	Li et al., 2019; Hofstra et al., 2000
F174	D2	Unknown	HSCR	Li et al., 2019
R175	D2	R175P	HSCR	Li et al., 2019; So et al., 2011
R180	D2	Unknown	HSCR	Li et al., 2019
C197	D2	C197Y	HSCR	Li et al., 2019; So et al., 2011
V202L	D2	V202L	HSCR	ClinVar VCV000599632.3
R231	D2	R231H	HSCR	Li et al., 2019; Pelet et al., 1998
E235	D2	E235K	HSCR	Hofstra et al., 2000
E251	D2	E251K	HSCR	Li et al., 2019; Kashuk et al., 2005
V262	D2	V262A	HSCR	Kashuk et al., 2005
D264	D3	D264K	HSCR	Pelet et al., 1998
D267	D3	D267N	HSCR	Kashuk et al., 2005
T278	D3	T278N	HSCR	ClinVar VCV000041846.35; Li et al., 2019
V282	D3	V282L	HSCR	Ruiz-Ferrer et al., 2006
R287	D3	Unknown	HSCR	ClinVar VCV000560625.3; Li et al., 2019
E289	D3	E289Q	HSCR	Kashuk et al., 2005
D300	D3	D300K	HSCR	Li et al., 2019; Pelet et al., 1998
R313	D3	R313Q	HSCR	Li et al., 2019; Kashuk et al., 2005; So et al., 2011
S316	D3	S316I	HSCR	Li et al., 2019; So et al., 2011
R330	D3	R330W/Q	HSCR	Li et al., 2019; Kashuk et al., 2005
W324	D3	W324C	HSCR	Kashuk et al., 2005
S339	D3	S339L	HSCR	Li et al., 2019; So et al., 2011
D353	D3	D353Y	HSCR	Li et al., 2019; So et al., 2011
N359	D3	Unknown	HSCR	Li et al., 2019
C570	D3	C570W	HSCR	Kashuk et al., 2005
R360	D3	R360Q	HSCR	Li et al., 2019; So et al., 2011
N361	D3	N361K	HSCR	Hofstra et al., 2000
P384	D3	P384W	HSCR	Nunez-Torres et al., 2011
F393	D3	F393L	HSCR	Li et al., 2019; Kashuk et al., 2005
N394	D3	Unknown	HSCR	Li et al., 2019
V397	D3	Unknown	HSCR	Li et al., 2019
P399	D3	P399L	HSCR	Li et al., 2019; Kashuk et al., 2005
V412	D4	V412M	HSCR	Li et al., 2019; So et al., 2011
G423	D4	G423R	HSCR	Li et al., 2019; So et al., 2011
L442	D4	L442P	HSCR	Nunez-Torres et al., 2011
D469	D4	D469N	HSCR	Anders et al., 2001
R475	D4	R475Q/W	HSCR	Li et al., 2019; Kashuk et al., 2005
E480	D4	E480K	HSCR	Li et al., 2019; So et al., 2011
C558	D5	C558Y	HSCR	Kim et al., 2006
Q576	D5	Q576P	HSCR	Hofstra et al., 2000
D584	D5	D584G	HSCR	Kashuk et al., 2005
C585	D5	C585R	HSCR	ClinVar VCV000695031.3
G588	D5	G588D	HSCR	Kashuk et al., 2005
E595	D5	E595G	HSCR	Li et al., 2019
F555	D5	F555C	**HSCR+MEN2A**	ClinVar VCV001777553.4; Li et al., 2019
C609	D5	C609R/S/G/F/Y/W	**HSCR+MEN2A**	**ME2A:** C609S – ClinVar VCV001372611.8; Wells et al., 2015 C609G – ClinVar VCV003254419.2 C609F – ClinVar VCV000038284.20; Wells et al., 2015 C609W – ClinVar VCV002497944.2 **HSCR+MEN2A:** C609G – Wells et al., 2015; C609R – ClinVar VCV000013944.17; C609Y – ClinVar VCV000013933.51; Wells et al., 2015, C609W – Pelet et al., 1998; Wells et al., 2015
C611	D5	C611R/S/G/F/Y/W	**HSCR+MEN2A**	**MEN2A:** C611S – ClinVar VCV001780971.5 C611G – ClinVar VCV000024897.12 C611W – ClinVar VCV000013913.7 C611Y – ClinVar VCV000024898.36 C611F – ClinVar VCV000024899.14 **HSCR+MEN2A:** C611S – Nishikawa et al., 2003; Wells et al., 2015 C611R – Clinvar VCV000024896.10; Virtanen et al., 2013
C618	D5	C618R/S/G/F/Y	**HSCR+MEN2A**	**MEN2A:** C618S – Clinvar VCV000013914.37 C618R – Clinvar VCV000013929.35 C618F – ClinVar VCV000024902.19 C618Y – ClinVar VCV000024901.24; Wells et al., 2015 **MEN2A+HSCR:** C618R – Decker and Peacock, 1998; Wells et al., 2015 C618S – Decker and Peacock, 1998; Wells et al., 2015 C618G – ClinVar VCV000013905.92
C620	D5	C620R/S/G/F/Y/W	**HSCR+MEN2A**	**MEN2A:** C620S – ClinVar VCV000038602.13 C620F – Clinvar VCV000013928.36 **HSCR+MEN2A:** C620R – ClinVar VCV000013915.41; Wells et al., 2015 C620G – ClinVar VCV000024905.22 C620S – Wells et al., 2015 C620W – ClinVar VCV000013934.15; Wells et al., 2015 C620Y – ClinVar VCV000013916.28
V292	D3	V292M	MEN2A	ClinVar VCV000024880.54; Castellone et al., 2010; So et al., 2011
G321	D3	G321R	MEN2A	Krampitz and Norton, 2014
C515	D5	C515S	MEN2A	Krampitz and Norton, 2014
C531	D5	Unknown	MEN2A	Li et al., 2019
G533	D5	G533C	MEN2A	ClinVar VCV000013950.30; Krampitz and Norton, 2014; Takahashi, 2022
C541	D5	C541Y	MEN2A	ClinVar VCV002133665.2
R600	D5	R600Q	MEN2A	Krampitz and Norton, 2014
K603	D5	K603E	MEN2A	Krampitz and Norton, 2014
Y606	D5	Y606C	MEN2A	Krampitz and Norton, 2014
C630	D5	C630R/S/F/Y	MEN2A	Li et al., 2019; Wells et al., 2015
C634	D5	C634R/S/G/F/W/Y	MEN2A	Takahashi, 2022; Li et al., 2019; Wells et al., 2015

77 RET positions, whose mutations are linked to HSCR, MEN2A or both.

Unknown, mutations to unknown amino acids are marked.

We further collected data on the clinical manifestations of MEN2A *RET* variants using information from the literature and ClinVar (Table 2). We categorized reported clinical manifestations according to the following clinical terms: medullary thyroid carcinoma (MTC), familial medullary thyroid carcinoma (FMTC), pheochromocytoma (PHEO), primary hyperparathyroidism (PHPT), cutaneous lichen amyloidosis (CLA) and Hirschsprung disease (HSCR). On the one hand, a couple of documented manifestations were found in RET domain D3, but most were in domain D5. MTC and FMTC are often manifested with PHEO and PHPT. On the other hand, CLA is exclusively associated with variants in position C634. HSCR was reported with detailed manifestations only in variants of positions F555, C609, C611, C618 and C620.

**Table 2.** Genotype-phenotype correlations for *RET* variants in MEN2A

RET variant	MTC	FMTC	PHEO	PHPT	CLA	HSCR	Source
**V292M**	** **	** **	**+**	** **	** **	** **	ClinVar VCV000024880.54; Castellone et al., 2010
**G321R**	** **	**+**	** **	** **	** **	** **	Wells et al., 2015
**C515S**	** **	**+**	** **	** **	** **	** **	Wells et al., 2015
**G533C**	+	** **	+	** **	** **	** **	Maciel et al., 2019
**F555C**		**+**		** **	** **	**+**	ClinVar VCV001777553.4; Li et al., 2019
**R600Q**		**+**					Krampitz and Norton, 2014
**K603Q**		**+**					Krampitz and Norton, 2014
**Y606C**		**+**					Krampitz and Norton, 2014
**C609R**	**+**	**+**	**+**	**+**		**+**	ClinVar VCV000013944.17; Kashuk et al., 2005; Maciel et al., 2019; Kim and Kim, 2021
**C609S**	**+**		**+**	**+**		**+**	ClinVar VCV001372611.8; Kashuk et al., 2005; Wells et al., 2015; Maciel et al., 2019; Kim and Kim, 2021
**C609G**	**+**		**+**	**+**		**+**	Wells et al., 2015; Maciel et al., 2019; Kim and Kim, 2021
**C609F**	**+**	**+**	**+**	**+**			Kim and Kim, 2021; Paszko et al., 2007
**C609Y**	**+**	**+**	**+**	**+**		**+**	ClinVar VCV000013933.51; Maciel et al., 2019; Kim and Kim, 2021
**C609W**		**+**	**+**			**+**	Kashuk et al., 2005; Maciel et al., 2019
**C611R**	**+**			**+**		**+**	Virtanen et al., 2013; Maciel et al., 2019
**C611S**	**+**		**+**	**+**		+	Wells et al., 2015; Maciel et al., 2019; Kim and Kim, 2021
**C611G**	**+**		**+**	**+**			Maciel et al., 2019; Kim and Kim, 2021
**C611F**	**+**	**+**	**+**	**+**			Kashuk et al., 2005; Kim and Kim, 2021
**C611R**	+		+				Wells et al., 2015; Maciel et al., 2019
**C611W**	**+**		**+**				Maciel et al., 2019
**C618R**		**+**	**+**	**+**		**+**	ClinVar VCV000013929.35; Kashuk et al., 2005; Maciel et al., 2019
**C618S**		**+**	**+**	**+**		**+**	ClinVar VCV000013914.37; Kashuk et al., 2005; Wells et al., 2015; Maciel et al., 2019
**C618G**		**+**				**+**	ClinVar VCV000013905.92
**C618F**	**+**		**+**	**+**			Maciel et al., 2019
**C618Y**		**+**					So et al., 2011
**C620R**	**+**	**+**	**+**	**+**		**+**	Kashuk et al., 2005; Virtanen et al., 2013; Maciel et al., 2019
**C620S**	**+**	**+**	**+**	**+**			Kashuk et al., 2005; Maciel et al., 2019
**C620G**	**+**		**+**	**+**			Maciel et al., 2019
**C620F**		**+**	**+**	**+**		**+**	ClinVar VCV000013928.41; Kim and Kim, 2021
**C620Y**	**+**						Huang et al., 2020
**C630R**	**+**		**+**	**+**			Wells et al., 2015; Kim and Kim, 2021
**C630S**		**+**					Wells et al., 2015
**C630F**		**+**					Wells et al., 2015
**C630Y**		+	+	+			Wells et al., 2015; Maciel et al., 2019; Kim and Kim, 2021
**C634R**	+		+	+	+		Maciel et al., 2019; Kim and Kim, 2021
**C634S**	+		+	+	+		Maciel et al., 2019; Kim and Kim, 2021
**C634G**	+		+	+	+		Maciel et al., 2019; Kim and Kim, 2021
**C634F**	+		+	+	+		Maciel et al., 2019; Kim and Kim, 2021
**C634W**	+		+	+	+		Maciel et al., 2019; Kim and Kim, 2021
**C634Y**	+		+	+	+		Maciel et al., 2019; Kim and Kim, 2021

Documented manifestations are marked with a plus (+) sign while blank cells indicate no mentions of specific manifestations in the literature. Abbreviations are as follows: MTC, medullary thyroid carcinoma; FMTC, familial medullary thyroid carcinoma; PHEO, pheochromocytoma; PHPT, primary hyperparathyroidism; CLA, cutaneous lichen amyloidosis; HSCR, Hirschsprung's disease.

We mapped 77 positions in Table 1 onto the 3D structure of the extracellular region of RET – classified as LOF, GOF or both (Fig. 1A). The 66 positions associated with LOF mutations are distributed across all five domains, but with fewer positions in D4 and in the CRD D5 (Fig. 1B). Notably, the five *RET* variants that can lead to both diseases are only in D5 (Fig. 1C) while GOF-only mutations are predominantly in the CRD D5–14 out of 16 (Fig. 1D). We noticed that almost all of the 16 GOF mutations (Fig. 1E) seem to be clustered in positions located in a specific region of CRD D5 (Fig. 1C,D).

### Predicted mechanistic principles for LOF effects in RET positions associated with HSCR

**Fig. 2.**
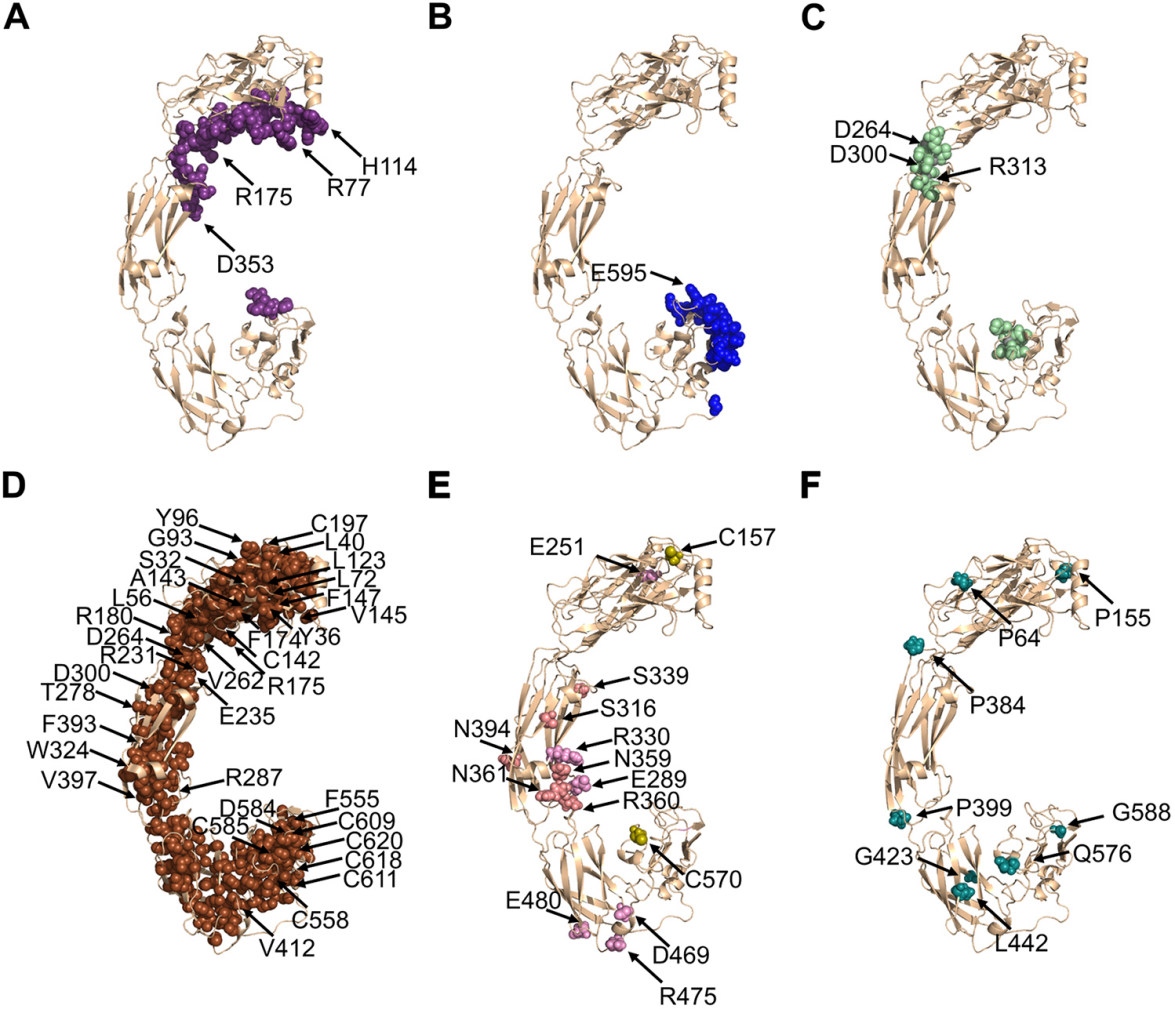
**Predicted structural basis for RET LOF mutations leading to HSCR disease.** (A) The 43 RET residues predicted to interact with RET co-receptors, shown as purple spheres. The four mutated positions among these residues that lead to HSCR are labeled with black arrows. (B) The 24 RET residues predicted to interact with RET ligands, shown as blue spheres. The one mutated position that leads to HSCR is labeled with a black arrow. (C) 19 RET residues that bind Ca^2+^, shown as light green spheres. The three mutated positions among these residues that lead to HSCR are labeled with black arrows. (D) The 222 RET residues classified as ‘buried’ in the protein core (rASA≤5% or BSA≥200 Å^2^), shown as brown spheres. The 36 mutated positions among these residues that lead to HSCR are labeled with black arrows. (E) The 14 RET residues that lead to HSCR and stabilize RET tertiary structure via strong intramolecular interactions are indicated by black arrows and shown as spheres colored light pink (participating in hydrogen bonds), pink (participating in salt bridges), and olive (forming intermolecular disulfide bonds). (F) Six RET positions that involve mutations to/from prolines/glycines, thereby, destabilizing RET tertiary structure, indicated by black arrows and shown as teal spheres.

We hypothesize that mutations that reduce downstream signaling by RET or that destabilize the protein and, thereby, reduce its amount in cells can lead to RET LOF and, thereby, to HSCR disease. Mutations in RET positions involved in protein−protein interactions with co-receptors or ligands may impair RET activation and reduce downstream signaling. Mutations that, by diverse mechanisms, destabilize RET, can also lead to LOF. One such mechanism may involve binding to calcium, as calcium ions (Ca^2+^) have been shown to stabilize RET tertiary structure (Li et al., 2019). Therefore, mutations in positions involved in Ca^2+^ binding may destabilize the receptor and disrupt its folding. Alternatively, substitutions for RET positions that are part of its protein core and stabilize its tertiary structure could also disrupt folding or structural integrity. Last, mutations in *RET* that disrupt strong intramolecular interactions may also affect its tertiary structure. Therefore, we mapped which residues mediate interactions between RET and its co-receptors and ligands, participate in the Ca^2+^-binding sites, are buried in the protein core, introduce a specific disruption of RET structures via amino acids with unique physicochemical properties, such as glycines or prolines, or may affect RET stability by disrupting strong intramolecular interactions (Fig. S1, Fig. 2).

We examined these 77 positions for effects on RET protein−protein interactions by analyzing the experimental structures of RET in complex with each of the following four co-receptors and four ligands, i.e. (1) RET with GFRα1 and GDNF (PDB ID 6Q2J), (2) RET with GFRα3 and artemin (PDB ID 6Q2S), (3) RET with GFRAL and GDF15 (PDB ID 6Q2N) and (4) RET with GFRα2 and neurturin (PDB ID 6Q2O), as well as the 4:4:4 structure of RET with GFRα2 and neurturin (PDB ID 6Q2R) (Li et al., 2019). RET residues located within 5 Å of the interface with co-receptors (Fig. 2A) or ligands (Fig. 2B) were classified as ’interacting residues’. We identified 43 RET residues within 5 Å of a co-receptor and 24 RET residues within 5 Å of a ligand (Fig. S2, Fig. 2A,B). RET residues that interact with co-receptors are mostly in domains D1 and D2, with only a few residues in D5. In contrast, RET residues that interact with ligands are only in domains D4 and D5. Among the 66 HSCR-associated RET positions that, presumably, reduce activity upon mutation, only four – i.e. R77, H114, R175 and D353 (Fig. 2A) – are in proximity to RET co-receptors, and only one position associated with HSCR – i.e. E595 – is within 5 Å of a ligand (Fig. 2B).

We next mapped positions involved in Ca^2+^ binding (Fig. 2C) since it has been shown to be important for RET stability (Li et al., 2019). Out of the 22 RET positions predicted to be involved in stabilizing the tertiary structure of RET through Ca^2+^ binding, two positions, D264 and D300, were associated with HSCR. A third HSCR-associated residue, arginine (R)313, is immediately adjacent to the Ca^2+^-binding Y314 and was, therefore, also classified as part of the Ca^2+^-binding site. Therefore, three of the 66 LOF RET positions participate in Ca^2+^ binding and likely lead to HSCR through destabilization of RET tertiary structure.

To map RET residues that are part of the core of the protein, we identified positions that are sufficiently buried in the protein hydrophobic core, where a mutation is more likely to perturb RET folding or destabilize the structure. To quantify the burial of each RET residue, we used an approach developed in our previous study (Bakhman et al., 2019). We measured the accessible surface area (ASA) of each RET residue using surfv (https://honig.c2b2.columbia.edu/surface-algorithms) (Nicholls et al., 1991). Then, we calculated the relative accessible surface area (rASA) for each residue by dividing the ASA value by the maximal empirical ASA value for each residue, with these ASA values taken from Tien et al. (2013). We also calculated the buried surface area (BSA) for each residue as the difference between the maximal empirical ASA value and the calculated ASA for each residue. We classified RET residues as ‘buried’ when either their rASA values were ≤15% or their BSA values were >200 Å^2^, with all other residues considered ‘exposed’. This burial-based analysis classified the 594 residues in the RET extracellular region as 222 residues being buried and 372 being exposed (Fig. 2D). We predicted that the 222 buried residues are more likely to destabilize RET tertiary structure upon mutations that change the physicochemical properties or size of the residue and, thereby, decrease RET levels in the relevant tissues. Indeed, 36 out of 66 RET LOF positions involve buried residues. We also note that two Ca^2+^-binding residues and one co-receptor-binding residue were also classified as buried, meaning that a mutation of these residues can lead to LOF due to multiple simultaneous causes. Taken together, 39 of the 66 LOF mutations in RET destabilized its tertiary structure by affecting the protein core and/or Ca^2+^ binding, and only four residues reduced its activity solely via protein−protein interactions with ligands or co-receptors. This left 23 RET positions not classified under the categories above.

Of these 23 RET positions associated with HSCR, 14 participate in intramolecular interactions that can affect RET stability and folding (Fig. 2E). Visual inspection identified that two of these 14 HSCR-associated RET positions (C157 and C570) are cysteine residues that participate in intramolecular disulfide bonds with residues C197 and C585, respectively. The other 12 RET residues participate in intramolecular hydrogen bonds or salt-bridges. Glutamic acid (E)251, serine (S)316 and E480 form intramolecular hydrogen bonds with R205, histidine (H)333 and threonine (T)503, respectively. R330 forms an intramolecular salt-bridge network with three negatively charged residues – E289, aspartic acid (D)290 and E332. Another salt-bridge is between D469 and R475, and two hydrogen bond networks are formed between S339, asparagine (N)359, R360 and N361, and between N394, glutamine (Q)371 and H392.

Eight of the remaining nine HSCR-associated RET positions involve changes either to or from proline or glycine (Fig. 2F). Such mutations are likely to affect RET stability because of the extreme backbone flexibility or rigidity associated with glycines or prolines, respectively (Levitt, 1978; Bajaj et al., 2007). P64 is in the middle of a loop next to a buried phenylalanine residue, F66. Consistent with previous findings (Anders et al., 2001), we predicted that the HSCR-associated P64L mutation may lead to steric interference with F66, thereby, affecting RET tertiary structure and stability. Similar to P64, P155 is in a loop and is also located between two buried residues, leucine (L)160 and tyrosine (Y)41. Likewise, D3 residues proline (P)339 and P384 are adjacent to the buried residues valine (V)400 and G383, respectively. Mutations in either of these prolines will likely perturb these regions in D3. Reciprocally, Q576 that is positioned in the middle of a loop in the D4-D5 interface and is adjacent to the buried residue glycine (G)453, and the D4 residue leucine (L)442 that is adjacent to the buried residue V485, are both substituted for a proline (P) residue in an HSCR-associated mutation. Thereby, these two variants will increase conformational rigidity and, presumably, negatively affect RET tertiary structure. In contrast, the G423R mutation inserts both a charge and a significant steric bulk next to two large residues, lysine (K)424 and Q421, thereby, likely perturbing RET tertiary structure. We note that five additional residues that were classified above as buried or involved in protein−protein interactions are also involved in mutations to or from prolines/glycines. Of these, L40P, L72P, G93S and D584P are mutations in buried residues, R175P is a mutation in a residue that is both buried and predicted to bind co-receptors, while E595P is a mutation in a residue predicted to bind ligands. Therefore, these positions can also contribute to HSCR through multiple distinct mechanisms.

Overall, our analysis revealed that, in 57 out of the 66 HSCR-associated LOF mutations, the involved RET residues are important to its folding or stabilize its tertiary structure, thus, providing mechanistic structural-based explanations to the association of these mutations with LOF/HSCR. Only four mutations appeared to contribute to HSCR by impacting solely protein−protein interactions. For only four HSCR-associated positions out of the 66 – i.e. R57, V202, D267 and V282 – our analysis did neither predict an effect on protein−protein interactions nor on protein stability. These were ‘exposed’ residues located in D2, D3 and D4, respectively, and were not predicted to interact with ligands or co-receptors. R57 is positioned in the middle of a loop, directly adjacent to proline and arginine residues. It is possible that the mutation of this charged arginine to a bulky tryptophan (Table 1) altered local intra-molecular interactions and, thereby, global stability. In contrast, V202, D267 and V282 are immediately adjacent to residues buried within the protein core. Specifically, V202 is immediately adjacent to the buried residue isoleucine (I)200, D267 is immediately adjacent to D266 and E178 and is also close to E265, while V282 is in direct contact with alanine (A)281 and V283. Several of these adjacent residues are buried yet charged residues, so it is plausible that these four *RET* variants will indirectly also affect adjacent buried residues and, thereby, RET tertiary structure and stability.

### Predicted mechanistic principles for RET variants associated with GOF effect and MEN2A

**Fig. 3.**
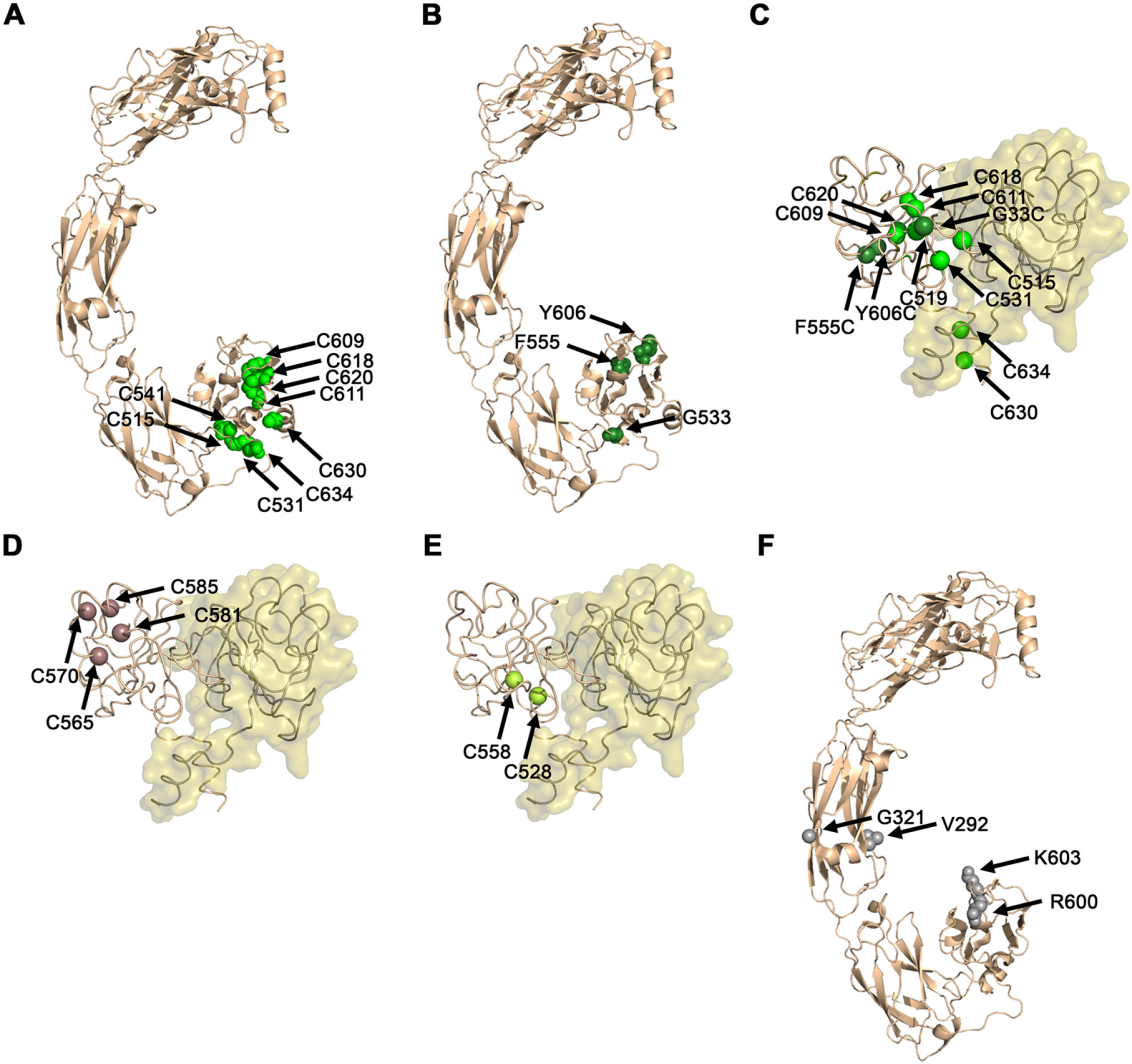
**Predicted structural basis for RET GOF mutations that lead to MEN2A.** (A) Nine cysteine residues that, upon mutation to a non-cysteine residue, result in an unpaired cysteine; all shown as green spheres. (B) Three non-cysteine residues that, upon mutation to a cysteine, also result in an unpaired cysteine; all shown as dark green spheres. (C) Positions of the 12 unpaired cysteines resulting from the mutations described in A and B, mapped onto one of the monomers in an Alphafold3 model of the D5−D5 dimer. Cα atoms of these unpaired cysteines are shown as spheres and colored as in A and B. Notice that eight of these 12 GOF mutations involve both cysteines across the same intramolecular disulfide bond. (D) Four cysteine residues in D5 that have not been associated with MEN2A, mapped onto one of the monomers in the model of the D5−D5 dimer (from C), with the Cα atoms shown as dark pink spheres. (E) Positions of the two cysteine residues in D5 that are close to the predicted D5−D5 interface but have not been associated with MEN2A, with their Cα atoms shown as light green spheres. (F) Four RET positions that lead to GOF upon mutation but do not involve changes to or from cysteines, shown as grey spheres.

Out of the 16 gain-of-function (GOF) positions identified in RET (Table 1), 12 mutations involved changes to or from cysteine residues that participate in disulfide bonds within the RET CRD D5. We hypothesized that these substitutions lead to an unpaired cysteine in D5, which can form an intermolecular disulfide bond with its unpaired counterpart across the RET dimer interface. Such an intermolecular disulfide bond will lead to aberrant RET homodimerization and, thus, to increased signaling that, presumably, underlies oncogenesis. Nine of these 12 variants changed a cysteine residue to a different amino acid residue (Fig. 3A). Interestingly, the remaining three of these 12 GOF variants – F555C, G533C and Y606C – also led to an unpaired cysteine but via a reciprocal route, i.e. instead of mutating half of an existing intramolecular disulfide bridge, they introduce a new unpaired cysteine residue (Fig. 3B).

In most cases of RET GOF that have been identified in patients, we found substitutions in both cysteines that span the same disulfide bond. However, in the disulfide bond between C541 and C519, only mutations in C541 were observed, leading to an unpaired C519 (Fig. 3C). As detailed above, all of these unpaired cysteines can, potentially, form intermolecular disulfide bonds with their counterparts across the dimer interface, similarly leading to RET homodimerization that can be independent of ligands and co-receptors. Indeed, we observed that all of the 12 unpaired cysteines that result from a GOF mutation are clustered around the same out-facing side of D5 (Fig. 3A,B), supporting the intermolecular dimerization hypothesis. To test this hypothesis, we modeled the D5−D5 dimer of RET by using AlphaFold3 (Abramson et al., 2024). This model showed that all 12 unpaired cysteine positions are in the region of D5 that faces the predicted homo-dimerization interface – 11 of the 12 unpaired GOF cysteine residues mentioned above are ≤9 Å from the modelled dimer interface, while F555C is ∼12 Å from this interface (Fig. 3C). In contrast, almost all cysteine residues in D5 that have not been associated with MEN2A were farther away from the predicted D5−D5 dimer interface, at distances of 13-18 Å (Fig. 3D). The only exception is C528, which is 7 Å from the predicted D5−D5 dimer interface (Fig. 3E); this residue forms an intramolecular disulfide bond with C558 but has not been reported to be associated with MEN2A. Therefore, this location suggests that a mutation of C558 may also lead to a GOF and to MEN2A.

The remaining four of the 16 reported GOF mutations did not lead to unpaired cysteines and, therefore, seem to contribute to MEN2A through a different mechanism (Fig. 3F). These mutations are in four positions, located in domains D3 (V292M, G321R) and D5 (R600W, K603E). The exposed residues V292 and G321 are far from both the ligand-binding and co-receptor-binding regions. The V292M mutation does not sufficiently change the physicochemical properties of this residue to explain the GOF phenotype. However, the G321R mutation, as it involves a dramatic change in the physicochemical properties of the residue, might induce an intramolecular conformational change that can increase RET activation. As for the two residues in D5, both R600 and K603 are close to the predicted D5−D5 dimer interface (Fig. 3C), and mutations of these positions might, therefore, also promote D5−D5 dimerization, albeit through a non-covalent mechanism that, probably, requires a conformational change. We note that K603 is within 5 Å of the co-receptor, but visual inspection did not identify RET/co-receptor interactions that might be enhanced as a result of this mutation and can explain the GOF phenotype. We also note that variants of the R600 residue, which is also positioned in close proximity to co-receptor-binding residues, have been reported to be extremely rare (Saez et al., 2000); so, perhaps the GOF phenotype is less pronounced. Overall, our analysis suggested that 14 of the 16 GOF mutations associated with MEN2A promote D5−D5 dimerization and, thereby, enhance downstream signaling and tumorigenesis.

### Predicted mechanistic explanation for the paradoxical LOF and GOF effects in RET Janus mutations associated with both HSCR and MEN2A

Five disease-associated RET positions – F555, C609, C611, C618 and C620 – are linked via Janus mutations to both HSCR and MEN2A (Table 1, Fig. 1). Our analysis revealed that all of these positions contribute to the stability of the tertiary structure of RET, as they are part of the protein core (Fig. 1D). Interestingly, it has been documented in patients that F555 is replaced exclusively by cysteine, while C609, C611, C618 and C620 are consistently changed to the same specific amino acids – arginine, serine, glycine, phenylalanine, tyrosine or tryptophan (Table 1). Therefore, all five Janus mutations involve substantial changes in physicochemical properties and are likely to affect protein folding. On the one hand, these substantial substitutions, particularly in such core residues, may destabilize the tertiary structure of RET, thereby impairing the embryonic development of the enteric nervous system and contributing to HSCR. On the other hand, these Janus mutations in all five residues, which are all located at the periphery of D5, always result in an unpaired cysteine (Fig. 3). F555C introduces a new and unpaired cysteine to the predicted D5−D5 interface, whereas the C609, C611, C618, and C620 positions are all part of intramolecular disulfide bonds, so their mutation leaves an unpaired cysteine close to the predicted D5−D5 interface. These unpaired cysteines can lead to aberrant RET dimerization as detailed above and, thereby, contribute to MEN2A that, unlike HSCR, manifests later in life.

## DISCUSSION

We curated a comprehensive dataset of 77 positions in the extracellular domains of RET that have been associated with either HSCR disease, MEN2A or both – integrating entries from both the ClinVar database and the literature (Tables 1-2). This dataset provides a reconciled and clarified view of *RET* variants that is more comprehensive than previously published and, to our knowledge, is the largest dataset of these disease-associated *RET* variants. We also provide predicted structural and mechanistic bases for the LOF and GOF phenotypes for almost all of these disease-associated mutations across these 77 RET positions. Our carefully curated dataset can serve as a benchmark for developing computational variant effect predictors (VEPs) (Gerasimavicius et al., 2025), which are promising as powerful tools for missense evaluation in the context of human health. Our detailed analysis of both GOF and LOF mutations also provides insights into how one should design such tools. For example, our analysis supports the common wisdom that LOF mutations tend to be broadly distributed, while GOF mutations show more spatial clustering (Gerasimavicius et al., 2025). Importantly, the RET example highlights that LOF and GOF mutations are not necessarily mutually exclusive.

Our structure-based analysis of this dataset suggests that ∼90% of RET extracellular positions associated with HSCR may destabilize the tertiary structure of the RET receptor. Only a small minority of mutations appear to cause LOF by attenuating protein−protein interactions with the co-receptors or ligands that activate RET. A previous analysis of disease-associated missense mutations across dozens of different proteins reached similar ratios, estimating ∼80% of such mutations impair protein stability, with the majority of these involving residues buried in the protein core or causing disruption of strong intramolecular interactions (Wang and Moult, 2001). Indeed, our predictions suggest that the most common reason for RET destabilization and, therefore, LOF is perturbation of the protein core. However, disruption of strong intramolecular interactions that include salt-bridges, hydrogen bonds and disulfide bonds or mutations at Ca^2+^-binding sites that were shown to stabilize RET tertiary structure, can also lead to LOF and HSCR disease. An additional mechanism for RET destabilization can occur when residues in the protein core or in intramolecular interfacial positions are changed to or from glycines or prolines, due to the unique effect of such mutations on protein 3D structure. Mutations involving glycine, with its extreme flexibility, or proline, which rigidifies the protein backbone and can adopt unique conformations, can disrupt local folding and global stability. Notably, some HSCR-associated positions can lead to LOF via two or even three different mechanisms. Specifically, mutations in D264 and D300 can destabilize RET not only by disrupting Ca^2+^ binding but also by compromising the protein core. Similarly, mutations in the buried residues L40, G93 and E584 can affect both the protein core due to their burial, or through the specific conformational effects of a change to or from glycine. Furthermore, the buried residue R175 is predicted to affect interactions with co-receptors but also involves a change to proline. Likewise, the E595P mutation is also predicted to affect ligand binding. Overall, we predict that mutations destabilizing the protein tertiary structure are the most likely to lead to HSCR.

In contrast to LOF mutations observed in HSCR, GOF mutations associated with MEN2A predominantly involve mutations to or from cysteine residues in the CRD D5 domain of RET. Such mutations lead to an unpaired cysteine close to the predicted D5−D5 dimer interface that presumably forms an intermolecular disulfide bond across the homodimer. This, in turn, leads to aberrant RET dimerization and activation that is less dependent on co-receptors and ligands. There are four cysteine residues in D5 that are not known to lead to GOF upon mutation, which our analysis suggests is due to their more distant location from the predicted dimer interface – presumably not allowing the formation of an intermolecular disulfide bond across the dimer interface. The additional GOF mutations that do not involve unpaired cysteines are also located in membrane-proximal domains, supporting a common GOF mechanism of enhancing RET homodimerization via these membrane-proximal domains. Two of these positions are located in D5, where they can enhance D5−D5 dimerization. A third position might involve activity-enhancing conformational changes, also supporting a general mechanism of GOF mutations leading to constitutive activation by enhancing RET dimerization proximal to the membrane. Indeed, these membrane-proximal interactions resemble other homotypic protein−protein interactions and, in particular, the juxta-membrane homodimerization shown to occur between other RTKs, i.e. PDGFR, KIT and VEGFR (Yuzawa et al., 2007; Yang et al., 2008, 2010). Finally, we predicted that variants in C558 can lead to an unpaired C528 – a mutation that has not been identified in patients to date – and, thus, contribute to MEN2A.

Our structural analysis suggests a mechanistic explanation for the, seemingly paradoxical, five RET Janus mutations that lead to both HSCR and MEN2A. Recent work has highlighted the complexity of RET Janus mutations, showing that mice carrying the RET C618F mutation in combination with a reduction in RET gene expression, developed intestinal aganglionosis due to aberrant RET activation (Okamoto et al., 2021). This, in turn, led to premature neuronal differentiation and impaired precursor migration. On the one hand, analysis showed that all Janus mutations are buried within D5 and that the substitutions found in HSCR involve dramatic changes to the physicochemical properties of these residues, explaining the LOF phenotype by a substantial destabilization of RET. On the other hand, all five mutations result in an unpaired cysteine, which is predicted to form a D5−D5 intermolecular disulfide bond, leading to the GOF phenotype as explained in detail above. The concurrent prediction, though, is that the LOF phenotype is partial, allowing sufficient levels of RET to facilitate abnormal dimer formation and MEN2A later in life. More broadly, the dual pathogenic phenotype of mutations in RET highlights the need for precisely tailored therapeutic strategies to address RET-associate diseases in the thyroid and enteric ganglia. Stabilizing the RET protein might help mitigate the LOF HSCR phenotype in the intestine, whereas preventing aberrant receptor activation is crucial for treating MEN2A. Furthermore, the pivotal role of cysteine residues within D5 in RET regulation underscores the potential of developing covalent inhibitors targeting intermolecular disulfide bonds as a therapeutic strategy for MEN2A.

## MATERIALS AND METHODS

### Protein structures

We used the following 3D structures in our analysis and visualization of RET with different co-receptors and ligands: RET with GFRα1 and GDNF (PDB ID 6Q2J), RET with GFRα3 and artemin (PDB ID 6Q2S), RET with GFRAL and GDF15 (PDB ID 6Q2N), RET with GFRα2 and neurturin (PDB ID 6Q2O), and 4:4:4 structure of RET with GFRα2 and neurturin (PDB ID 6Q2R). Short segments of RET (residues 129-137, 207-211, 246-251 and 379-387) that are missing in PDB entries 6Q2R, 6Q2O, 6Q2S and 6Q2S were modeled using Loopy (Xiang et al., 2002) and partial or missing side chains were modeled using Scap (https://honig.c2b2.columbia.edu/scap) (Xiang et al., 2002). We used the Alpahfold3 server (https://alphafoldserver.com/) (Abramson et al., 2024) to predict the 3D structure of the RET D5−D5 dimer and PyMol (https://www.pymol.org/) for structural visualization.

### Structure-based prediction of mutation effect

RET residues ≤5 Å of the interface with co-receptors and ligands were classified as ‘interacting residues’. Ca^2+^-binding residues were classified according to Li et al. (2019). To map residues that can affect the tertiary structure of a protein due to being buried in the protein core, we followed the methodology described previously (Bakhman et al., 2019). We measured the accessible surface area (ASA) of each residue using surfv (https://honig.c2b2.columbia.edu/surface-algorithms) (Nicholls et al., 1991). Then, we calculated the relative accessible surface area (rASA) for each residue by dividing its ASA by the maximal empirical ASA, taken from the values calculated from a large dataset of structures culled from the PDB in (Tien et al., 2013), who followed the approaches laid out by Rose et al. (1985) and Miller et al. (1987). Buried surface area (BSA) was calculated by subtracting the ASA of each residue from the maximal empirical ASA value for that residue. A residue was classified as ‘buried’ when its rASA ≤15% or when its BSA ≥200 Å^2^. Residues involved in intramolecular salt bridges or hydrogen bonds were identified by visual inspection.

## Supplementary Material

10.1242/dmm.052748_sup1 Supplementary information
