# Supplementary material for: Systematic structure-based analysis of RET variants in MEN2A and Hirschsprung's disease, and the paradoxical co-occurrence of both conditions
Source: Dis Model Mech. 2026 Apr 27;19(4):dmm052748. doi: 10.1242/dmm.052748 (PMC13225206; doi:10.1242/dmm.052748)
Supplement: Supplementary information [file dmm-19-052748-s1.pdf]

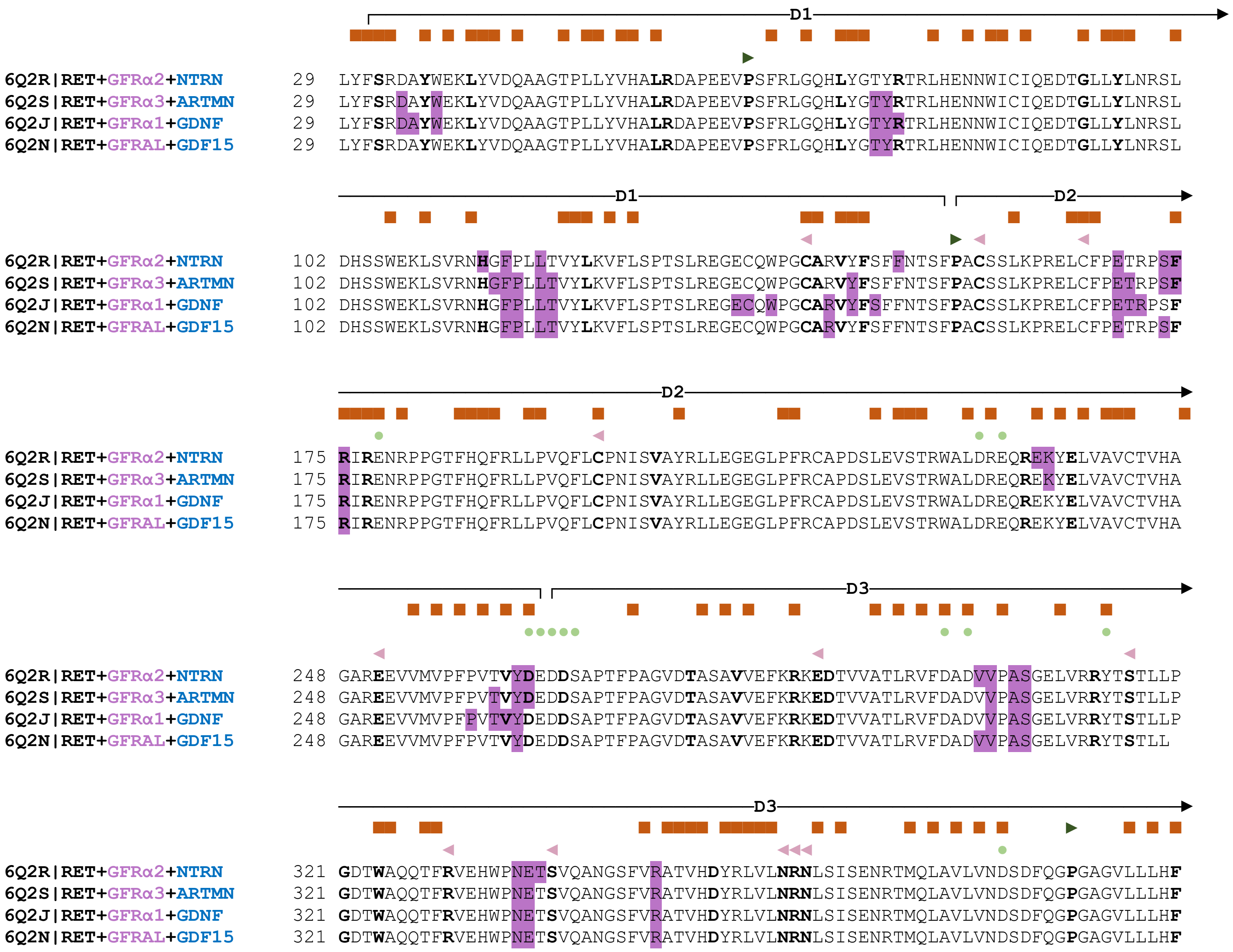


Disease Models & Mechanisms • Supplementary information

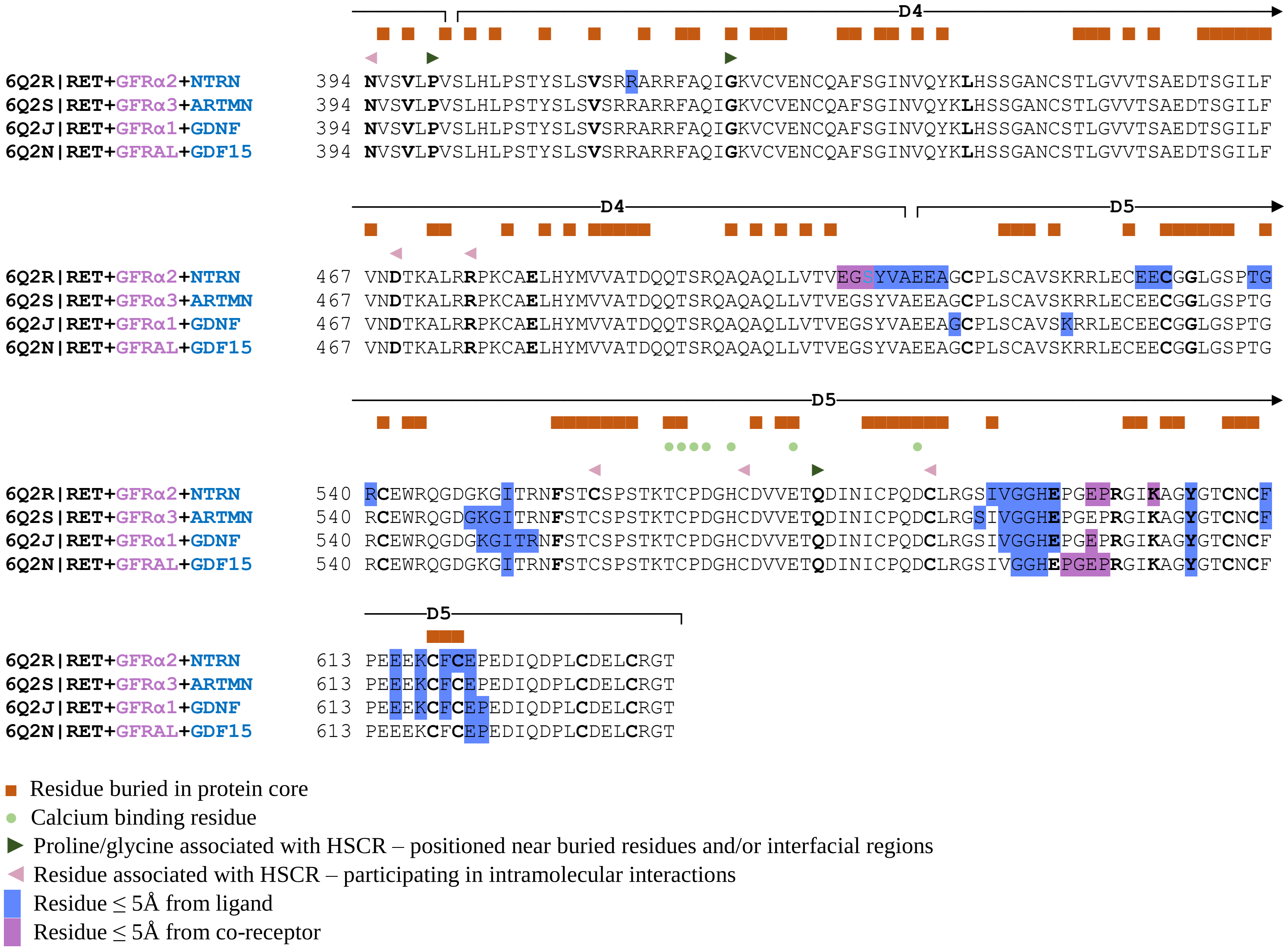


**Fig. S1. Residues in RET structures that may affect protein tertiary structure or intermolecular interactions.** Residues that can affect tertiary structure, participate in intramolecular interactions, or in protein-protein interactions with ligands or co-receptors are annotated above the alignment according to the key. RET positions associated with MEN2A or HSCR diseases are marked in bold.

Disease Models & Mechanisms • Supplementary information

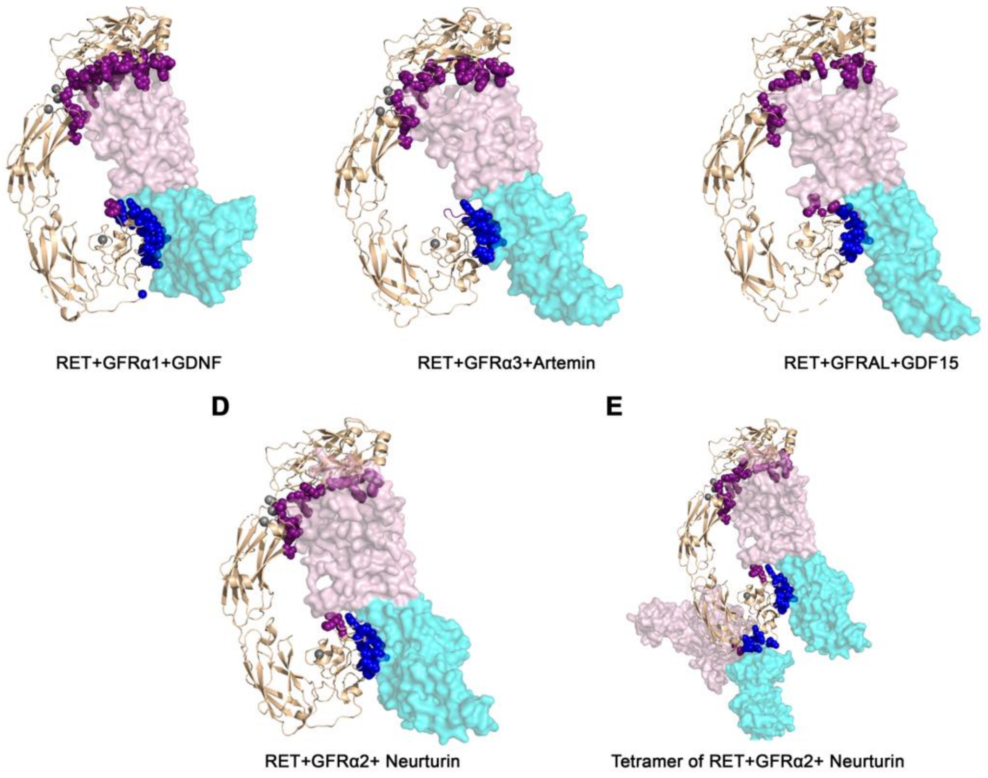


**Fig. S2. Residues in the extracellular region of RET ≤ 5Å from ligands or co-receptors. A.** Residues in RET (wheat ribbon) ≤ 5Å of the GFRα1 co-receptor (pink molecular surface) or the GDNF ligand (cyan molecular surface) in PDB ID 6Q2J, shown as spheres and colored blue and purple, respectively. **B.** Residues in RET ≤ 5Å of the GFRα3 co-receptor (pink molecular surface) or the Artemin ligand (cyan molecular surface) in PDB ID 6Q2S, shown as in A. **C.** Residues in RET ≤ 5Å of the GFRAL co-receptor (pink molecular surface) or the GDF15 ligand (cyan molecular surface) in PDB ID 6Q2N, shown as in A. **D.** Residues in RET ≤ 5Å of the GFRα2 co-receptor (pink molecular surface) or the Neurturin ligand (cyan molecular surface) in PDB ID 6Q2O, shown as in A. **E.** Residues in RET ≤ 5Å of the two GFRα2 co-receptors (pink molecular surface) or the two Neurturin ligands (cyan molecular surface) in the RET tetramer complex in PDB ID 6Q2R, shown as in A.
